# Forecasting and Evaluating Multiple Interventions for COVID-19 Worldwide

**DOI:** 10.3389/frai.2020.00041

**Published:** 2020-05-22

**Authors:** Zixin Hu, Qiyang Ge, Shudi Li, Eric Boerwinkle, Li Jin, Momiao Xiong

**Affiliations:** ^1^State Key Laboratory of Genetic Engineering and Innovation Center of Genetics and Development, School of Life Sciences, Fudan University, Shanghai, China; ^2^Human Phenome Institute, Fudan University, Shanghai, China; ^3^The School of Mathematic Sciences, Fudan University, Shanghai, China; ^4^School of Public Health, The University of Texas Health Science Center at Houston, Houston, TX, United States

**Keywords:** COVID-19, artificial intelligence, transmission dynamics, forecasting, time series, auto-encoder

## Abstract

As the Covid-19 pandemic surges around the world, questions arise about the number of global cases at the pandemic's peak, the length of the pandemic before receding, and the timing of intervention strategies to significantly stop the spread of Covid-19. We have developed artificial intelligence (AI)-inspired methods for modeling the transmission dynamics of the epidemics and evaluating interventions to curb the spread and impact of COVID-19. The developed methods were applied to the surveillance data of cumulative and new COVID-19 cases and deaths reported by WHO as of March 16th, 2020. Both the timing and the degree of intervention were evaluated. The average error of five-step ahead forecasting was 2.5%. The total peak number of cumulative cases, new cases, and the maximum number of cumulative cases in the world with complete intervention implemented 4 weeks later than the beginning date (March 16th, 2020) reached 75,249,909, 10,086,085, and 255,392,154, respectively. However, the total peak number of cumulative cases, new cases, and the maximum number of cumulative cases in the world with complete intervention after 1 week were reduced to 951,799, 108,853 and 1,530,276, respectively. Duration time of the COVID-19 spread was reduced from 356 days to 232 days between later and earlier interventions. We observed that delaying intervention for 1 month caused the maximum number of cumulative cases reduce by −166.89 times that of earlier complete intervention, and the number of deaths increased from 53,560 to 8,938,725. Earlier and complete intervention is necessary to stem the tide of COVID-19 infection.

## Introduction

As of March 16th, 2020, the number of confirmed cases of COVID-19 worldwide surpassed 170,568, and the occurrence has spread to more than 152 countries. As this coronavirus has become classed as a pandemic (Callaway, 2020), a number of questions have arisen among the populous as well as government and business leaders: How many cases will there be worldwide? How many deaths can be expected? When will a peak in the number of cases occur? When will this pandemic end? How will the recommended immediate action slow the spread?

A number of statistical and dynamic models of COVID-19 outbreaks, including the SEIR model and branching processes, have been previously applied to analyze its transmission dynamics (Hellewell et al., 2020; Kucharski et al., 2020; Tuite and Fisman, 2020; Wu et al., 2020; Zhao et al., 2020; Li Q. et al., 2020). These epidemiological models are useful for estimating the dynamics of transmission, targeting resources, and evaluating the impact of intervention strategies, but the models require values for unknown parameters and depend on many assumptions (Funk et al., 2018; Johansson et al., 2019; Li R. et al., 2020).

Most analyses used hypothesized parameters and hence do not fit the data very well. The accuracy of forecasting the future cases of Covid-19 using these models may not be very high. The non-pharmaceutical interventions (NPIs) that attempt to reduce the reproduction number are the major strategies to curb the spread of Cvid-19. The NPIs include home quarantine, keeping social distancing, stopping mass gatherings, and the closure of schools and universities. We can simulate the effect of each single intervention. However, it is difficult to associate each single intervention with the real data. The intervention strategies that have been developed by these models cannot be evaluated by real data. Only comprehensive interventions can be associated with the real data.

To overcome limitations of the epidemiological model approach and assist public health planning and policy making, we developed the modified auto-encoder (MAE) (Yuan et al., 2018; Charte et al., 2019), an artificial intelligence (AI)-based method for real-time forecasting of the new and cumulative confirmed cases of Covid-19 worldwide and evaluating the impact of the comprehensive public health interventions and their implementation times on curbing the spread of Covid-19. The MAE does not consider single intervention but can model mandatory and voluntary comprehensive public health interventions while still using real data for evaluation of interventions.

Transfer learning was used to train the MAE (Zhuang et al., 2019). An intervention variable was introduced as an input variable for the MAE. We viewed the China type of intervention as the fully comprehensive intervention and assigned 1 to the intervention variable. We assigned 0 to the intervention variable if there was no intervention. The weights between 0 and 1 were assigned to the intervention variable for the different degrees of interventions. The values that were assigned to the intervention variable was called weight. Taking time for intervention into account, we considered different comprehensive intervention scenarios. We investigated how the degree of intervention and starting intervention time determine the peak time and case ending time, the peak number and maximum number of cases, and the forecast for the peak and maximum number of new and cumulative cases in more than 152 countries across the world. The analysis is based on the surveillance data of confirmed and new Covid-19 cases worldwide up to March 16th, 2020.

In this study, we aimed to develop an AI -nspired method for real-time forecasting and evaluation of the impact of comprehensive interventions on the curbing the spread of Covid-19 and show that earlier and complete intervention is necessary to stem the tide of COVID-19 infection. We estimated the maximum number of cumulative cases under earlier complete intervention to be 1,530,276; under later intervention the number of cases increased to a frightening 255,392,154, the number of deaths increased from 53,560 to 8,938,725, and the case ending time was significantly delayed. We concluded that, if there is no immediate aggressive action to intervene, we will face serious consequences.

## Materials and Methods

### Modified Auto-Encoder for Modeling Time Series

**Figure 1:**
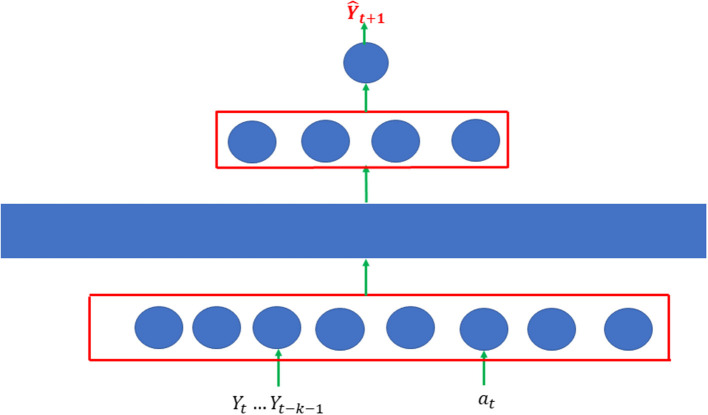
Architecture of modified autoencoder which consisted of two single AE. Each single AE was a three-layer feedforward neural network.

The MAE were used to forecast the number of the accumulative and new confirmed cases of Covid-19 and evaluate the impact of the comprehensive public health interventions on the spread of Covid-19. Unlike the classical auto-encoder where the number of nodes in the layers usually decreases from the input layer to the latent layers, the numbers of the nodes in the input, the first latent layer, the second latent layer, and the output layers in the MSAE were eight, 32, four, and one, respectively (Figure 1).

MAE consisted of two single AE. Each single AE was a three-layer feedforward neural network. The first layer is the input layer, the third layer is the reconstruction layer, and the second layer is the hidden layer. The input vector is denoted by $X_{t}={\left[Y_{t},\;Y_{t-1},\ldots ,\;Y_{t-k-1},\;a_{t}\right]}^{T}$, where *Y*_*t*_ is the number of cases at the time *t*, and 0 ≤ *a*_*t*_ ≤ 1 is the public health intervention indicator variable. If there is no intervention, then *a*_*t*_ = 0. For the strongest intervention, 1 is assigned to the variable *a*_*t*_ = 1. The input vector is mapped to the hidden layer to capture the features of the transmission dynamics of Covid-19 with public health intervention:

$$\begin{array}{l} h_{t}=\sigma_{1}\left(W_{hx}X_{t}+b_{h}\;\right), \end{array}$$

where *h*(*X*) is the hidden vector, *W*_*hx*_ are the weights connecting the input vector to the hidden layer, *b*_*h*_ is a bias vector, and σ_1_ is element-wise non-linear activation function ReLU.

AE attempts to generate an output that reconstructs its input by mapping the hidden vector to the reconstruction layer:

$$\begin{array}{l} {\tilde{X}}_{t}=\sigma_{2}\left(W_{oh}h_{t}+b_{o}\right), \end{array}$$

where $\tilde{X}$ is the output, *W*_*oh*_ are the weights connecting hidden layer to the output layer, *b*_*o*_ is a bias vector, and σ_2_ is element-wise non-linear activation function ReLU. The single layer AE attempts to minimize the error between the input vector and the reconstruction vector. The loss function is defined as

$$\begin{array}{l} l_{t}=\sum_{n=1}^{n}\sum_{t=k}^{T}||X_{t}^{n}-{\tilde{X}}_{t}^{n}|{|}^{2}\;. \end{array}$$

We develop stacked autoencoders with four layers that consist of two single-layer AEs stacked layer by layer [1]. The dimensions of the input layer, the first hidden layer, and the second hidden layer are eight, 32, and four, respectively (Figure 1). The first single-layer autoencoder maps the input vector into the first hidden vector by minimizing the reconstruction errors via gradient descent algorithm (Charte et al., 2019). After the first single-layer AE was trained, we removed the reconstruction layer of the first single layer AE and kept the hidden layer of the first single AE as the input layer of the second single- layer AE. In other words, the input vector of the subsequent AE was the hidden vector of the previous AE [1]. We repeated the training process for the second single-layer AE. The output of the final node that fully connects to the hidden layer of the second single-layer AE was the predicted number of cases Ŷ_*t*_*n*__ = *f*(*H*_*n*_) for the *n*^*th*^ sample, where *H*_*n*_ is the hidden vector of the second single-layer AE for the *n*^*th*^ sample. Our goal was to make the predicted Ŷ_*n*_ as close to the observed *Y*_*n*_ as possible. The loss function for prediction is

$$\begin{array}{l} l_{p}=\sum_{n=1}^{N}W_{n}||{Ŷ}_{t_{n}}-Y_{t_{n}}|{|}^{2}, \end{array}$$

where weight *W*_*n*_ will be defined in Data-preprocessing Section.

An intervention variable was introduced as an input variable for the MAE. We viewed the China-type intervention as the fully comprehensive intervention and assigned 1 to the intervention variable. We assigned 0 to the intervention variable if there was no intervention. Weights between 0 and 1 were assigned to different degrees of interventions—zero being no intervention and one being complete—including social distancing, hand washing, wearing face mask, strict travel restriction, no large group gatherings, mandatory quarantine, restricted public transportation, closing schools, and closure of all non-essential businesses, including manufacturing. We considered four intervention scenarios, which were described in Table S2. For each scenario, we investigated how the degree and timing of the intervention determined the peak and case-ending time, the number of cases at the peak, and the maximum number of cases.

### Data Pre-processing

We considered 152 time series (number of new cases collected for each day)—one time series for each country. The data were organized in a matrix with the rows representing the country and columns representing the number of the new confirmed cases of each day. Let *m* be the number of days. Let *t*_*ij*_ be the number of the confirmed new cases of the *j*^*th*^ day within the *i*^*th*^ country. Let *Z* be a 152 × *m* dimensional matrix. The element *Z*_*ij*_ is the number of the confirmed new cases of Covid-19 on the *j*^*th*^ day—starting with January 20th, 2020—in the *i*^*th*^ country.

One time series for the country in the training set was divided into a *k* = 44 subsegment of time series, each subsegment of time series with the number of new cases in 8 successive days. We viewed a subsegment of time series with 8 days as a sample of data.

One element from the data matrix *Z* is randomly selected as a start day of the subsegment and select its 7 successive days as the other days to form a subsegment of time series. Let *i* be the index of the time series and *j*_*i*_ be the column index of the matrix *Z* that was selected as the starting day. The *sub*segment of time series can be represented as {*Z*_*j*_*i*__,, *Z*_*j*_*i*_+1_, …, *Z*_*j*_*i*_+7_}. Data were normalized to $X_{j_{i}+k}=\frac{Z_{j_{i}+k}}{S},\;k=0,\;1,\;\ldots ,7$, where $S=\frac{1}{8}\sum_{k=0}^{7}Z_{j_{i}+k}$. Let $Y_{j_{i}}=\frac{Z_{j_{i}+8}}{S}$ be the normalized number of new cases to forecast. If *S* = 0, then set *Y*_*j*_*i*__ = 0. The *j*_*i*_ started with 9 and ended with *k*+8, the last day for the training, where *k* is the number of subsegments. The loss function was defined as

$$\begin{array}{l} L=\sum_{i=1}^{152}\sum_{j_{i=9}}^{k+8}W_{j_{i}}(Y_{j_{i}}-{Ŷ}_{j_{i}}{)}^{2}, \end{array}$$

where *Y*_*j*_*i*__ was the observed number of the new cases in the forecasting day of the ${j_{i}}^{th}$ subsegment time series, and Ŷ_*j*_*i*__ was its forecasted number of new cases by the MAE, and *W*_*j*_*i*__ were weights. If *j*_*i*_ was in the interval [1, 12], then *W*_*i*_ = 1. If *j*_*i*_ was in the interval [13, 24], then *W*_*i*_ = 2, etc. The back-propagation algorithm was used to estimate the weights and bias in the MAE. Repeat training processed five times. The average forecasting Ŷ_*j*_*i*__, *i* = 1, …, 152 will be taken as a final forecasted number of the confirmed new cases for each country.

### Forecasting Procedures

To forecast each day, we needed to take a matrix of the data that consisted of a subsegment of time series (number of new cases with 8 days) from each country and denoted the number of new cases in the *j*^*th*^ day for the *i*^*th*^ country by *x*_*i*_*j*__ . The trained MAE was used for forecasting the future number of new cases of Covid-19 for some day (*j*^*th*^ day) in the each country. Consider the *i*^*th*^ country. Assume that the number of new confirmed cases of Covid-19 on the *j*^*th*^ day that needs to be forecasted is *x*_*ij*_. Let *H* be a 152 × 8 dimensional matrix that was used for forecasting, *h*_*il*_ = *x*_*ij*−9+*l*_, *i* = 1, …, 152, *and l* = 1, …, 8 . Let $g_{i}=\frac{1}{8}\sum_{l=1}^{8}h_{il},\;i=1,\ldots ,\;152$ be the average of the *i*^*th*^ row of the matrix *H*. Let *U* be the normalized matrix of *H*, where $u_{il}=\frac{h_{il}}{g_{i}},\;i=1,\ldots ,\;152,\;and\;l=1,\ldots ,8$. The output of the MAE is the forecasted number of new confirmed cases and is denoted as ${\hat{v}}_{i}=f\left(u_{i1},\ldots .,\;u_{i8,\;}\theta \right),\;i=1,\ldots ,152$ , where θ represents the estimated parameters in the trained MAE. The one-step forecasting of the number of new confirmed cases of Covid-19 for each country is given by Ŷ_*i*_ $={\hat{v}}_{i}g_{i},\;i=1,\ldots ,\;152.$

The recursive multiple-step forecasting involved using a one-step model multiple times where the prediction for the preceding time step was used as an input for making a prediction on the following time step. For example, to forecast the number of new confirmed cases for the next day, the predicted number of new cases in one-step forecasting were used as observational input in order to predict day 2. The above process was then be repeated to obtain the two-step forecasting. The summation of the final forecasted number of new confirmed cases for each country was taken as the prediction of the total number of new confirmed cases of Covid-19 worldwide.

### Data Collection

The analysis is based on surveillance data of confirmed cumulative and new COVID-19 cases worldwide as of March 16th, 2020. Data on the number of cumulative and new cases and COVID-19-attributed deaths across 152 countries from January 20th to March 16th, 2020, were obtained from WHO (https://www.who.int/emergencies/diseases/novel-coronavirus-2019/situation-reports).

## Results

### Later Intervention Makes It Difficult to Stop the Spread of COVID-19

To demonstrate that the MAE is an accurate forecasting method, the MAE was applied to confirmed accumulated cases of COVID-19 across 152 countries. The intervention indicator for China and other countries was set to 1 and 0, respectively. Table 1 presents the one- to five-step errors for forecasting cumulative number of cases starting from March 12th, 2020. In all scenarios, the average forecasting accuracies of the MAE were <2.5% (Table 1). Table S1 presented the one- to five-step errors for forecasting cumulative number of cases of Covid-19 in China using MAE and ARIMAX, starting from March 4th, 2020. The maximum of average errors of one- to 5-step forecasting using MAE and ARIMAX was 0.0195% and 0.625%, respectively. The forecasting accuracy of MAE was much smaller than that of ARIMAX.

**Table 1:** One- to five-step forecasting errors.

	**Reported**	**1-step predicted**	**1-step errors (%)**	**2-step error (%)**	**3-step error (%)**	**4-step error (%)**	**5-step error (%)**
3/12/2020	125774	126272	0.40				
3/13/2020	133774	130278	−2.61	0.32			
3/14/2020	143864	144715	0.59	−3.58	−0.10		
3/15/2020	155618	153628	−1.28	2.51	−4.08	0.03	
3/16/2020	170568	163932	−3.89	−2.02	2.26	−4.80	0.34
Average absolute error			1.75	2.11	2.15	2.42	0.34

**Figure 2:**
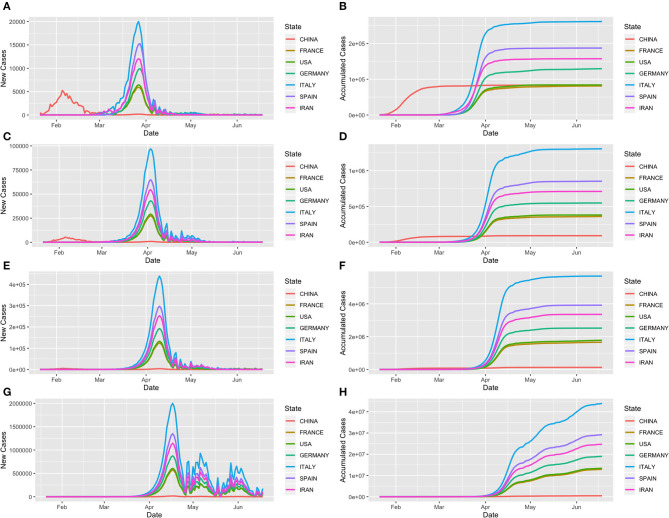
Trajectory of COVID-19 in the seven most infected countries—Iran, Spain, Italy, Germany, USA, France and China as a function of days from January 21st to June 19th, 2020. **(A,C,E,G)** Forecasted curves of the newly confirmed cases of COVID-19 under scenarios 1, 2, 3, and 4, respectively. **(B,D,F,H)** Forecasted curves of the cumulative confirmed cases of COVID-19 under scenarios 1, 2, 3, and 4, respectively.

Table 2 shows the forecasting results of COVID-19 in 30 countries and worldwide under a later stepwise intervention scenario (Scenario 4). The worldwide cumulative number of cases and the number of new cases at the peak with later intervention could reach 75,249,909 and 10,086,085, respectively. If every country in the world undertook such a later intervention scenario, the total number of cases in the world could reach as high as 255,392,154, and the community transmission of COVID-19 would continue until January 10th, 2021. The top 10 countries with a high average number of cases were Italy, Spain, Iran, Germany, USA, France, Switzerland, Belgium, UK, and Austria. To show the dynamics of COVID-19 development, Figures 2G,H shows the curves of the number of cumulative cases and new cases in seven major infected countries: Iran, Spain, Italy, Germany, USA, France, and China under scenario 4.

**Table 2:** Spread of Covid-19 in 30 countries worldwide under 4 weeks delay intervention.

**State**	**Peak time**	**End time**	**Duration**	**Peak (cum)**	**Peak (new)**	**Current case**	**End case**
Total	4/17/2020	1/10/2021	356	75249909	10086085	170568	255392154
Italy	4/17/2020	1/10/2021	346	14945480	1999429	24747	53281848
Spain	4/17/2020	1/10/2021	345	10080564	1351788	7753	33196999
Iran	4/17/2020	1/6/2021	322	8556153	1146663	14991	27343905
Germany	4/17/2020	1/10/2021	349	6532219	875856	4838	21864400
USA	4/17/2020	1/10/2021	356	4532725	607493	4740	16644849
France	4/17/2020	1/10/2021	352	4263429	572051	5380	14555999
Swizterland	4/17/2020	1/10/2021	320	3092785	414952	2200	9772913
Belgium	4/17/2020	1/5/2021	336	2835657	380783	1085	8727195
UK	4/17/2020	1/10/2021	345	1624266	218542	1395	6349494
Austria	4/17/2020	1/10/2021	320	1156505	156173	959	4206694
Norway	4/17/2020	1/10/2021	319	1214800	163068	1077	3894919
Malaysia	4/17/2020	1/10/2021	351	1081414	144904	553	3750555
Greece	4/17/2020	11/4/2020	252	1047665	141301	331	3595859
Netherlands	4/17/2020	1/10/2021	318	881147	118402	1135	3080802
Portugal	4/17/2020	1/10/2021	314	675964	91093	245	2104149
Finland	4/17/2020	1/10/2021	347	578886	77668	267	1923049
Estonia	4/17/2020	1/10/2021	319	607872	81796	205	1902652
Slovenia	4/17/2020	1/10/2021	312	598294	80475	219	1891314
Israel	4/17/2020	1/10/2021	324	526864	71296	200	1867519
Canada	4/17/2020	1/10/2021	350	480352	64450	304	1792760
Czechia	4/17/2020	1/8/2021	313	500323	67284	298	1708210
Iceland	4/17/2020	1/10/2021	315	438161	59381	138	1570527
Romania	4/17/2020	1/10/2021	319	383176	51910	158	1389549
Qatar	4/17/2020	1/10/2021	316	428531	57690	401	1245999
Brazil	4/17/2020	1/6/2021	315	374378	50246	200	1218993
Australia	4/17/2020	1/10/2021	352	353747	47491	298	1190874
Korea	4/17/2020	10/30/2020	284	296036	38849	8236	1019408
Poland	4/17/2020	1/5/2021	308	287008	38725	150	985182

### New Strategies Are Needed to Curb the Spread of COVID-19

There is an urgent need to develop new strategies to curb the spread of COVID-19 (Callaway, 2020). We investigated whether early complete interventions would reduce the peak time, cumulative case numbers, and the final total number of cases worldwide. Table 3 shows the forecasted results of COVID-19 in 30 countries and worldwide under early complete intervention (Scenario 1). We observed dramatic reduction in the number of COVID-19 cases. The forecasted total number of cases worldwide was reduced by early complete intervention to 1,530,276 from nearly 255 million with later intervention (Scenario 4). In other words, 99.4% of the potential cases could be eliminated by early complete intervention. The duration time was reduced from 356 days to 232 days, and the end time changed from January 10th, 2021, to September 8th, 2020. Figures 2A,B plot curves of the number of cumulative cases and new cases in six major infected countries—Iran, Spain, Italy, Germany, USA, and France—under Scenario 1.

**Table 3:** Spread of Covid-19 in 30 countries and worldwide under early complete intervention (1 week from March 16th intervention).

**State**	**Peak time**	**End time**	**Duration**	**Peak (cum)**	**Peak (new)**	**Current case**	**End case**
Total	2020/3/28	2020/9/8	232	951799	108853	170568	1530276
Italy	2020/3/27	2020/9/8	222	161276	19998	24747	261790
Spain	2020/3/28	2020/8/20	202	117400	15268	7753	187157
Iran	2020/3/27	2020/6/14	116	95104	12039	14991	157269
Germany	2020/3/28	2020/7/22	177	73998	9933	4838	129654
USA	2020/3/27	2020/6/6	138	47058	6454	4740	83921
China	2020/2/5	2020/4/29	100	31432	5236	81077	83103
France	2020/3/27	2020/8/2	191	45186	5933	5380	81593
Swizterland	2020/3/28	2020/9/6	194	34665	5031	2200	61734
Belgium	2020/3/28	2020/6/4	121	29479	4487	1085	52925
UK	2020/3/28	2020/6/2	123	19348	2467	1395	31006
Norway	2020/3/28	2020/6/22	117	13631	1985	1077	26386
Austria	2020/3/29	2020/6/5	101	14394	1825	959	24550
Greece	2020/3/29	2020/6/10	105	13525	1922	331	22467
Malaysia	2020/3/28	2020/7/4	161	11271	1705	553	20985
Netherlands	2020/3/26	2020/5/13	76	8097	1232	1135	16080
Korea	2020/2/29	2020/5/22	123	3150	813	8236	15649
Portugal	2020/3/30	2020/6/2	92	8578	1157	245	14841
Finland	2020/3/30	2020/7/22	175	7707	1037	267	13817
Estonia	2020/3/30	2020/6/21	116	7928	1036	205	13382
Slovenia	2020/3/28	2020/6/28	116	5856	958	219	12717
Israel	2020/3/29	2020/6/30	130	5865	878	200	10838
Iceland	2020/3/29	2020/6/23	114	4854	800	138	10679
Czechia	2020/3/28	2020/6/20	111	5653	772	298	9586
Canada	2020/3/28	2020/7/8	164	5330	739	304	9282
Qatar	2020/3/29	2020/6/11	103	4794	652	401	8206
Romania	2020/3/29	2020/5/21	85	4158	627	158	7754
Australia	2020/3/28	2020/6/13	141	4117	585	298	7430
Brazil	2020/3/28	2020/6/11	106	4017	584	200	7162
Denmark	2020/3/12	2020/6/19	114	615	353	898	6083

To investigate intervention measures between early complete and a 4-week delay intervention, Tables 4, 5 show the results under scenarios 2 and 3, respectively. Figures 2C–F plot transmission dynamics of COVID-19 with curves of the cumulative cases and new cases in the six major infected countries under scenarios 2 and 3, respectively.

**Table 4:** Spread of Covid-19 in 30 countries and worldwide under 2 weeks delay intervention.

**State**	**Peak time**	**End time**	**Duration**	**Peak (cum)**	**Peak (new)**	**Current case**	**End case**
Total	4/3/2020	9/11/2020	235	3657852	493023	170568	6522982
ITALY	4/3/2020	9/8/2020	222	727996	96948	24747	1307179
Spain	4/3/2020	8/20/2020	202	477245	64939	7753	852807
Iran	4/3/2020	7/23/2020	155	413873	54653	14991	710755
Germany	4/3/2020	7/23/2020	178	309434	43000	4838	549478
USA	4/3/2020	9/2/2020	226	216943	29222	4740	381178
France	4/3/2020	8/18/2020	207	204820	27442	5380	363355
Swizterland	4/3/2020	9/6/2020	194	145504	20435	2200	257680
Belgium	4/3/2020	7/3/2020	150	132216	19337	1085	237907
UK	4/3/2020	9/11/2020	224	77356	10561	1395	138340
Norway	4/3/2020	6/22/2020	117	57561	8410	1077	105766
Austria	4/4/2020	7/23/2020	149	62095	7971	959	103793
Malaysia	4/4/2020	7/4/2020	161	57868	7303	553	93940
China	2/5/2020	6/6/2020	138	31432	5236	81077	91305
Greece	4/3/2020	7/20/2020	145	48448	6958	331	88863
Netherlands	4/3/2020	8/2/2020	157	42387	5724	1135	75051
Portugal	4/3/2020	6/22/2020	112	30116	4706	245	59791
Slovenia	4/3/2020	7/15/2020	133	27465	4190	219	56030
Estonia	4/3/2020	7/20/2020	145	27602	4221	205	55039
Finland	4/4/2020	7/22/2020	175	31047	4302	267	53472
Israel	4/4/2020	6/30/2020	130	27678	3705	200	45801
Czechia	4/3/2020	6/20/2020	111	23470	3259	298	41366
Canada	4/3/2020	7/8/2020	164	22704	3198	304	40782
Iceland	4/4/2020	6/23/2020	114	22483	3087	138	37996
Brazil	4/3/2020	7/9/2020	134	17542	2509	200	34112
Romania	4/4/2020	6/15/2020	110	19969	2759	158	33605
Qatar	4/3/2020	6/11/2020	103	18725	2701	401	33116
Korea	4/5/2020	5/31/2020	132	24100	1873	8236	31670
Australia	4/3/2020	7/3/2020	161	16648	2310	298	29334
Poland	4/3/2020	6/13/2020	102	13287	1908	150	24239
Indonesia	4/4/2020	7/28/2020	149	12137	1811	117	23177

**Table 5:** Spread of Covid-19 in top 30 countries and worldwide under 3 weeks delay intervention.

**State**	**Peak time**	**End time**	**Duration**	**Peak (cum)**	**Peak (new)**	**Current case**	**End case**
Total	4/10/2020	12/4/2020	319	16528763	2221889	170568	29313739
Italy	4/10/2020	9/8/2020	222	3278431	439028	24747	5693059
Spain	4/10/2020	8/20/2020	202	2206610	297488	7753	3919623
Iran	4/10/2020	8/21/2020	184	1882888	252756	14991	3360378
Germany	4/10/2020	8/31/2020	217	1426977	192760	4838	2521231
USA	4/10/2020	10/10/2020	264	992158	133015	4740	1801181
France	4/10/2020	9/23/2020	243	933029	124787	5380	1674855
Swizterland	4/10/2020	9/6/2020	194	677240	92147	2200	1210668
Belgium	4/10/2020	12/4/2020	304	620500	85031	1085	1114935
UK	4/10/2020	9/11/2020	224	354043	47385	1395	639280
Norway	4/10/2020	8/2/2020	158	266353	36481	1077	477043
Austria	4/10/2020	7/23/2020	149	251230	34028	959	446229
Malaysia	4/10/2020	8/7/2020	195	235384	32064	553	417237
Greece	4/10/2020	8/23/2020	179	227442	30714	331	404882
Netherlands	4/10/2020	11/8/2020	255	192602	25705	1135	353763
Portugal	4/10/2020	7/5/2020	125	147451	20653	245	263904
Estonia	4/10/2020	8/4/2020	160	132648	18392	205	239417
Slovenia	4/10/2020	9/1/2020	181	130582	18060	219	236385
Finland	4/10/2020	8/30/2020	214	125404	17387	267	221710
Israel	4/10/2020	8/13/2020	174	113848	15554	200	203392
Czechia	4/10/2020	7/7/2020	128	109112	14697	298	194123
Canada	4/10/2020	10/17/2020	265	104602	14127	304	184096
Qatar	4/10/2020	8/19/2020	172	94166	13163	401	172432
Iceland	4/10/2020	7/20/2020	141	94695	13174	138	167504
Romania	4/10/2020	9/17/2020	204	82758	11451	158	147628
Brazil	4/10/2020	7/10/2020	135	81666	11128	200	145770
Australia	4/10/2020	7/6/2020	164	77308	10457	298	138540
China	2/5/2020	6/18/2020	150	31432	5236	81077	127241
Korea	4/10/2020	7/29/2020	191	70196	8899	8236	123488
Poland	4/10/2020	8/24/2020	174	62393	8447	150	111894
Egypt	4/10/2020	8/10/2020	178	58876	8110	126	106174

### Comparisons Among Intervention Strategies

**Figure 3:**
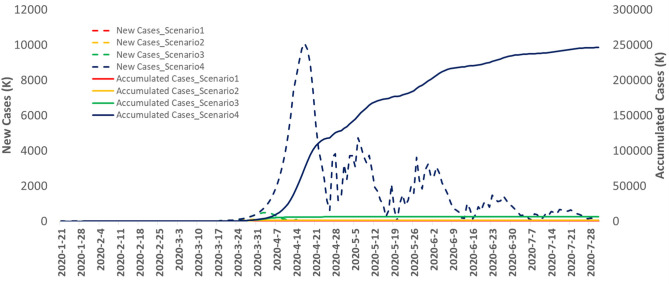
The reported and forecasted curves of the cumulative and new confirmed cases of Covid-19 in the world as a function of days from January 20th, to July 28th, 2020.

To further illustrate the impact of interventions on the spread of COVID-19, we compared the effects of four intervention scenarios on the transmission dynamics of COVID-19 across the world. Figure 3 plots the worldwide reported and forecasted time curves of the cumulative and newly confirmed cases of COVID-19 under the four intervention scenarios. The ratios of the world number of final cases across the four scenarios were 1:4.26:19.16:166.9, and the ratios of case duration under the four intervention scenarios were 1:1:01.1.38:1.53. These results demonstrate that intervention time delays have serious consequences.

**Figure 4:**
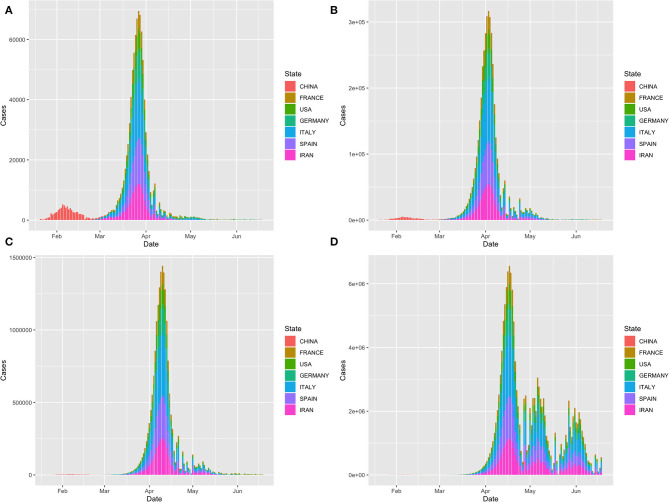
Time-case plot of the top seven infected countries: Iran, Spain, Italy, Germany, USA, France and China. **(A)** Time-case plot under intervention scenario 1; **(B)** Time-case plot under intervention scenario 2; **(C)** Time-case plot under intervention scenario 3 and **(D)** Time-case plot under intervention scenario 4.

Figure 4 plots the time-case curves for the top six infected countries: Iran, Spain, Italy, Germany, USA, France, and China. The time-case curve under the 4 week delay intervention was shifted more than 1 month to the right and was much steeper than that of under the early intervention. Delaying intervention will substantially increase the number of cumulative cases of COVID-19.

**Figure 5:**
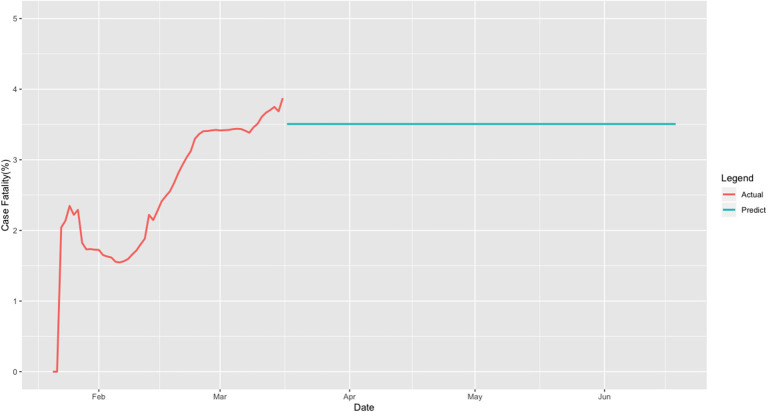
Case-fatality curve for the world.

Figure 5 shows the case-fatality rate curve as a function of time, where the case-fatality rate is defined as the ratio of the number of deaths over the number of cumulative cases in the world. The average case-fatality rate was 3.5%.

### Comparison With the SEIR Epidemiological Model

To illustrate the performance of the MAE for forecasting the transmission dynamics of COVID-19, we compared the MAE with the widely used epidemiological models. The susceptible-exposed-infected-recovered (SEIR) model is a standard mathematical compartmental model based on the average behavior of a population under study (Sameni, 2020). We compared the results of MAE for forecasting the peak time, peak number of new cases, and the maximum number of the cumulative cases of COVID−19 in China with a modified SEIR epidemiological model (Yang et al., 2020). The estimated peak time and peak number of new cases using the MAE method were February 5th, 2020, and 5,236, respectively. The estimated peak time and peak number of new cases using the modified (SEIR) epidemiological model were February 7th, 2020, and 4,169, respectively. The reported numbers of new cases from February 5th, 2020, to February 9th, 2020, in the WHO dataset were 5,229, 4,947, 4,158, 4593, and 3,534. It was clear that the peak time was February 5th, 2020. The MAE method precisely estimated peak time. The error of forecasting the peak number of new cases using the MAE method and modified SEIR model were 0.00134 and −0.203, respectively. The estimation using the MAE was much accurate than using the modified SEIR model.

The estimated maximum numbers of cumulative cases without inflow from abroad using the MAE and modified SEIR model were 83,103, and 122,122, respectively. The reported number of cumulative cases on May 2nd, 2020, was 84,338. The errors of forecasting the maximum number of cumulative cases using the MAE and modified SEIR model were −0.015 and 0.447, respectively. Again, the MAE substantially outperformed the modified SEIR model for forecasting the maximum number of cumulative cases of COVID-19 in China.

## Discussion

As an alternative to the epidemiologic transmission model, we used MAE to forecast the real-time trajectory of the transmission dynamics and generate the real-time forecasts of Covid-19 across the world. The results showed that the accuracies of prediction and subsequently multiple-step forecasting were high. This approach allows us to address two important questions: Is comprehensive NPIs required or not? How important is the intervention time? Since interventions are complicated and are difficult to quantify, we designed four intervention scenarios to represent the degrees of interventions and delay of interventions. The proposed methods combine the real data and some assumptions. This allowed us to evaluate the consequences of intervention while keeping the analysis as close to the real data as possible.

The MAE models allow us to input the interventions information, investigate the impact of interventions on the size, duration, and time of the virus outbreak, and recommend the intervention time.

Our results showed that real-time forecasting is more accurate than epidemiologic transmission model where the model parameters may not be applicable in practice. We estimated the duration, peak time, ending time, peak number, and maximum number of cumulative cases of COVID-19 under four intervention scenarios for 152 countries in the world. The forecasted total number of cases worldwide was reduced by early complete intervention to 1,530,276 from nearly 255 million with later intervention. In other words, 99.4% of the potential cases could be eliminated by early complete intervention. A delay of 4 weeks will substantially speed the spread of coronavirus, delay the ending time by almost 4 months, and increase the number of deaths from 53,560 to 8,938,725. These data provide critical information for government leaders and health authorities to consider urgent public health response to slow the spread of Covid-19. We have demonstrated that aggressive intervention is urgently needed.

## Data Availability Statement

These data can be downloaded from WHO Coronavirus disease (COVID-2019) situation reports at https://www.who.int/emergencies/diseases/novel-coronavirus-2019/situation-reports.

## Author Contributions

ZH performed data analysis. QG assist data analysis. SL pre-processing data. EB wrote paper. LJ designed project. MX designed project and wrote paper.

## Conflict of Interest

The authors declare that the research was conducted in the absence of any commercial or financial relationships that could be construed as a potential conflict of interest.
