# Supplementary material for: Forecasting and Evaluating Multiple Interventions for COVID-19 Worldwide
Source: Front Artif Intell. 2020 May 22;3:41. doi: 10.3389/frai.2020.00041 (PMC7861333; doi:10.3389/frai.2020.00041)
Supplement: Supplementary file 1 [file Table_1.docx]

| Table S1. One- to five-step forecasting errors using MAE and ARIMAX. | | | | | | | |  |  |  |  |
| --- | --- | --- | --- | --- | --- | --- | --- | --- | --- | --- | --- |
|  |  | MAE | | | | | ARIMAX | | | | |
|  | Reported | Estimated | | | | | | | | | |
|  | China | 1-Step | 2-Step | 3-Step | 4-Step | 5-Step | 1-Step | 2-Step | 3-Step | 4-Step | 5-Step |
| 3/4/2020 | 80422 | 80378 |  |  |  |  | 80353 |  |  |  |  |
| 3/5/2020 | 80565 | 80508 | 80557 |  |  |  | 80552 | 80353 |  |  |  |
| 3/6/2020 | 80711 | 80621 | 80577 | 80696 |  |  | 80761 | 80675 | 80353 |  |  |
| 3/7/2020 | 80813 | 80713 | 80647 | 80668 | 80873 |  | 80890 | 80967 | 80798 | 80353 |  |
| 3/8/2020 | 80859 | 80756 | 80718 | 80696 | 80736 | 81017 | 80824 | 81046 | 81198 | 80920 | 80353 |
|  |  | Errors | | | | | | | | | |
| 3/4/2020 |  | 1-Step | 2-Step | 3-Step | 4-Step | 5-Step | 1-Step | 2-Step | 3-Step | 4-Step | 5-Step |
| 3/4/2020 |  | 0.054% |  |  |  |  | 0.085% |  |  |  |  |
| 3/5/2020 |  | 0.071% | 0.009% |  |  |  | 0.016% | 0.263% |  |  |  |
| 3/6/2020 |  | 0.112% | 0.166% | 0.018% |  |  | 0.062% | 0.044% | 0.443% |  |  |
| 3/7/2020 |  | 0.123% | 0.206% | 0.179% | 0.074% |  | 0.095% | 0.190% | 0.018% | 0.569% |  |
| 3/8/2020 |  | 0.127% | 0.174% | 0.202% | 0.152% | 0.195% | 0.043% | 0.231% | 0.419% | 0.076% | 0.625% |
| Average |  | 0.098% | 0.139% | 0.133% | 0.113% | 0.195% | 0.060% | 0.182% | 0.293% | 0.322% | 0.625% |
