# Supplementary material for: Forecasting and Evaluating Multiple Interventions for COVID-19 Worldwide
Source: Front Artif Intell. 2020 May 22;3:41. doi: 10.3389/frai.2020.00041 (PMC7861333; doi:10.3389/frai.2020.00041)
Supplement: Supplementary file 2 [file Table_2.docx]

Table S2. Description of four scenarios of interventions.

| Scenario | Description |
| --- | --- |
| Scenario 1 | One week after the March 16 with a weight of 0.5, one week later, it transitioned to an intervention with a weight of 1. |
| Scenario 2 | Started with a weight of 0 for the first week, a weight of 0.5 for the second week, and two weeks later transitioned to a complete intervention with a weight of 1. |
| Scenario 3 | Intervention was delaying two weeks, a weight of 0.5 for the third week and finally transitioned to complete intervention with a weight of 1 in the fourth week |
| Scenario 4 | Delayed actions for three weeks, a limited intervention with a weight of 0.5 for the fourth week and finally transition to complete intervention with a weight of 1. |
